# On the education of the orthopedic resident

**DOI:** 10.4103/0019-5413.41847

**Published:** 2008

**Authors:** Augusto Sarmiento

**Affiliations:** University of Miami School of Medicine, Miami, Fl, USA

It is only logical to suspect that a discussion on the education of the orthopedic resident may be influenced by personal experiences, which can adversely affect the objectivity necessary to draw sound and impartial conclusions. My analysis of the situation is no exception.

I wrote a few years ago that “despite the many developments in the medical sciences during the past few decades the education of the orthopedist had retained most of its traditional format. The adaptive changes that it had experienced may not have been as significant as they could or should have been”.^1^ Today, I propose to look more carefully into the current scenario and analyze the challenges created not only by the evolution of the profession, but also by societal behavioral forces.

In the United States, medical care has undergone profound changes regarding its delivery and cost. The latter has reached major proportions, to the point that radical changes loom on the horizon. The growing number of uninsured people, reaching 47 million, and the huge difference in the care the poor and the financially better off receive, has prompted a clamor for the establishment of a Universal Health Insurance, which until now had been successfully opposed by special interests' parties that would be financially affected by its implementation.

These phenomena are the necessary backdrop that require recognition when attempting to discuss education matters. The seminal and uncontroversial reality is that Medicine is rapidly ceasing to be a profession, and becoming a business. The values of medicine are being replaced with those of the business community. The unselfish commitment that professionals are supposed to give to those they serve is vanishing from the everyday life of the physician. Profit is replacing the altruism that characterized the medical art for many a generation and now it appears to be its *raison d'etre.*

## INDUSTRY'S ROLE IN EDUCATION

I have long believed that the crescendo of the harmful change commenced when the pharmaceutical and surgical implant industry began to get control of the education of the orthopedist. This control was not gained overnight. It was a gradual and calculated effort, which within less than one generation reached the degree where it can be said that “The education of the orthopedist is structured primarily for the purpose of satisfying the marketing needs of the industry”.^1^–^3^

It is hypocritical to deny the above reality. The dominance of the industry in the life of the orthopedic surgeon is overwhelming. Through thousands of “educational courses” directly or indirectly subsidized by the industry, the student is bombarded with information regarding new and/or improved techniques that primarily benefit the manufacturers of the products. That many of these products have advanced the care of the orthopedically disabled is unquestionable. It is the unreasonably expensive price of the products and the abuse committed by the surgeons that have caused the damage.

This abuse, reaching the level of corruption, is frequently committed by orthopedists at the urging and manipulation concocted by the industry. This problem has now reached the ears of the public from revelations exposed by the media. The use of kickbacks to orthopedists in tens and hundreds of thousands of dollars for the use and support of industrial products has come to the attention of the Justice Department, which at this time is investigating gross transgressions in the relationship between the orthopedic community and the industry.

Evidence is also mounting about the pernicious influence of the industry in support of research, indicating that the veracity of studies conducted under the aegis of the industry is highly questionable.

Needless to say, the ongoing preference for the surgical option in the management of virtually all musculoskeletal conditions is frequently based on economic considerations. Hospitals prefer surgical patients because they profit from the higher charges they submit to insurance companies and additional laboratory tests. Likewise, the tremendous differences in the reimbursement orthopedic surgeons receive from surgical versus non-surgical care drives the trend. Unaware of these not so subtle features, the resident in training grows believing that the choice of surgical care selected by his mentors is based on scientific, logical reasons rather than economic benefits.^4^

This explains why it is no longer possible to believe that we are “educating” scientists/surgeons. Rather, we are “training” cosmetic surgeons of the skeleton; skeletal cosmetologists. Residents learn that any deviation from the normal, e.g. a resulting mild shortening of an extremity, or a few degrees of angulation upon completion of healing of a fracture in a long bone, or any degree of joint incongruity is a complication that the scalpel must overcome.

The economic survival of many educational programs has become almost totally dependent on the support the industry gives to residency programs and other educational organizations. The funded visiting professors, lecturers, traveling for faculty and residents alike, as well as the conduct of research are clear examples.

## THE CURRENT ENVIRONMENT

It is in this unhealthy environment that many residents are currently educated. Most of them are unaware of the greed that underlies some of the popular practices in their respective programs.^5^–^7^ They assume that what they read or are told by their mentors is based on appropriately documented scientific evidence, and do not know that the encouragement to perform surgery is oftentimes done for the simple purpose of enriching their coffers. Examples abound: how can anyone with a modicum of basic ethical principles, knowledge and experience claim that all fractures of the clavicle, wrist, metacarpals and phalanges should be treated surgically? That surgical replacement of an arthritic condition readily amenable to symptomatic treatment is the treatment of choice, or that a mildly painful back condition in the elderly, secondary to degenerative changes that are controllable with symptomatic medications must be treated with major surgery? It is greed what drives the promoters of such unethical, radical and extreme practices to openly advocate them. Not only do some of them receive large amounts of money for the promotion, but they also personally benefit from the higher reimbursements that surgical modalities offer at this time.

For as long as Medicine is a business, it will not be possible to create a sound educational system. Sooner or later, a solution will be imposed by outside powers, much to the detriment of the traditional autonomy that sustained our profession for so many years.^2^,^6^,^7^

In the United States the Residency Accreditation Body has done a good job over the years trying to monitor compliance with established requirements. However, since the reviews are cursory and carried out within a very short period of time, verification of the data submitted to the reviewer cannot be guaranteed. There are institutions with major flaws who escape the criticism they deserve. Their number, fortunately, is small.

In some busy programs with major responsibilities for the care of the indigent, it is not uncommon to see residents serve only as retractor-holders for the duration of rotations through some subspecialties. For example, I have heard residents seeking fellowships in spine surgery for which they suspect there is a national unwritten conspiracy to perpetuate that practice in order to minimize competition as much as possible. In those programs, Fellows are the only ones allowed to learn the surgical techniques implemented by their mentors. This policy is irresponsible and must be condemned.

In some programs, residents assist the faculty in the operating room, without ever having had the chance to see the patients prior to surgery. Subsequently, they take care of monitoring the in-hospital course, change dressings and plan the patient's discharge. However, they are not given the opportunity to see long follow-ups, since attendance to the attending surgeons' offices is not permitted.

## EXAGGERATED FRAGMENTATION

The fragmentation of orthopedics into smaller structured subspecialties appeared at first glance to be a logical and healthy development, since subspecialization is a natural phenomenon in virtually all human endeavors. I embraced the trend with enthusiasm when it first began to explode. Personally, I made plans to structure the two departments of Orthopedics over which I had the privilege to preside at the Universities of Miami and the University of Southern California. Within a short time I had on their faculty representatives from virtually all existing subspecialties. However, within a relatively short time, I began to realize that exaggerated subspecialization could harm the educational process.

As a result of the fragmentation into a very large number of subspecialties, residency programs have seen themselves composed of numerous individuals in their faculties whose practices and areas of teaching are limited to small fragments of the profession. These individuals enhance the caliber. They expect, and often receive, block times for their educational endeavors. Residents are assigned to them for specific periods of time. Since it is not unusual to see departments with divisions in Foot and Ankle surgery, Hand Surgery, Shoulder and Elbow Surgery, Hip and Knee surgery, Sports Medicine, Spine surgery, Children Orthopedics, Musculoskeletal Infections, Orthopedic Oncology, Research and Trauma, it should be obvious that the overall time devoted to each section may be too short for all the participating residents to obtain sufficient knowledge and surgical skills to qualify them to appropriately take care of most musculoskeletal conditions upon entering into the rough-and-tumble of private practice. The rotations through all these sections could be shortened to fit into the five years of training. Fortunately, some programs combine some of the rotations resulting in longer periods of exposure to the conditions that fall into those categories.^1^,^7^,^8^

## POSTGRADUATE FELLOWSHIPS

The above described system gave birth to post-residency fellowships; a trend that eventually reached universal proportions. At this time, medical students applying for residency positions are already committed to taking a fellowship following completion of the five years of residency. Their reasons often defy logical thinking. Most of them mistakenly assume that without a fellowship training their chances of finding a satisfactory position in the community are jeopardized. Others do it simply because “everybody does it”.

It is ironic that the choice of fellowship is frequently based not on perceived deficiencies in a specific area, but in areas that happened to be the most popular and glamorous at the time. Currently, sports medicine is the darling of the crowd, having replaced hand surgery and joint replacement.

The large number of fellowship-trained orthopedists will eventually reach a saturation point; a phenomenon that has already manifested itself in some cities. Such saturation implies that many newly graduated fellows will not be able to practice in the area in which they spent an additional year. They might end up indulging in areas where they did not feel sufficiently comfortable at the end of their residency days.

Hoping to market themselves in the community, they must overcome hurdles such as membership in subspecialty societies that call for additional examinations, and the payment of membership fees. Meeting those unnecessary requirements does not ensure success.^9^

The reader may have already surmised that I have serious reservations about the manner in which fellowships are structured today. Though I readily agree that some residents benefit from postgraduate fellowships, not all of them profit from them. Residents who graduate from well-balanced programs do not need them. Upon completion of their training they should be sufficiently competent to handle the vast majority of musculoskeletal problems. The clinical conditions with which they do not feel comfortable, they voluntarily eliminate from their practices and refer them to others. This is the way orthopedics was practiced for many a generation.

The length of fellowships need not be standardized as being always of one year. Three or six months are sometimes sufficient to ensure gaining the additional desired knowledge. As an example, for the resident who at the completion of his/her years of training has been directly involved in the care of all traumatic, infectious or degenerative conditions in the upper extremity, the spending of an additional year observing hundreds of carpal tunnel releases and open reduction of fractured wrists and metacarpals is a waste of time. Three to six months would be more than enough.

The exaggerated fragmentation of Orthopedics into such large numbers of subspecialties [26 subspecialties are officially recognized by the American Academy of Orthopedics as members of the Council of Specialty Societies (CMSS)], has unwillingly contributed to the erosion of our profession; an event that had a major impact in residency education. Orthopedics has lost within the last two generations a large segment of its territory, as exemplified by the greater involvement of Plastic Surgeons in the care of fractures and reconstructive skeletal surgery in the upper extremity, the neurosurgeons taking care of a large proportion of spine surgery stabilization; the podiatrist becoming what the public considers the true foot surgeons, and others.^2^,^7^

## RESEARCH

It has been recommended that residents should be directly involved in research for specific periods of time during the five years of training. The concept is sound if for no other reason than involvement in research gives the resident a closer appreciation of the complexity of investigative studies. However, I doubt the value of compulsory rotations through research. Many residents are not at all interested in such an experience, which takes them away from clinical activities. On the other hand, residents who are interested in research should be given as much time as possible to indulge in it.^5^,^10^

## CONCLUSIONS

In summary, I believe that the education of the orthopedic resident in the United States can be improved in some regards. Whether a similar perception can be extended to other countries is impossible for me to suggest. The inclusion of separate time-blocks for too many subspecialties makes some rotations too short to make them worthwhile. Merging some of them could be beneficial. Exaggerated emphasis on postgraduate fellowships for every resident might soon prove counterproductive. Some residents benefit form them but the majority do not need them. The loss of control of education by the profession and its current control by the pharmaceutical and surgical implant industry underlines the many problems we are experiencing today. The conversion of medicine from a profession into a business and the resulting loss of professionalism in our ranks, which has become rather severe in recent times, must be overcome if we are to restore the dignity that characterized medicine for many a generation.^2^,^4^,^5^,^7^
